# Neuronal Delamination and Outer Radial Glia Generation in Neocortical Development

**DOI:** 10.3389/fcell.2020.623573

**Published:** 2021-02-05

**Authors:** Ayano Kawaguchi

**Affiliations:** Department of Anatomy and Cell Biology, Nagoya University Graduate School of Medicine, Nagoya, Japan

**Keywords:** neuronal delamination, Lzts1, neural progenitor cell, outer radial glial cell, adherens junction, AKNA, neocortical development

## Abstract

During neocortical development, many neuronally differentiating cells (neurons and intermediate progenitor cells) are generated at the apical/ventricular surface by the division of neural progenitor cells (apical radial glial cells, aRGs). Neurogenic cell delamination, in which these neuronally differentiating cells retract their apical processes and depart from the apical surface, is the first step of their migration. Since the microenvironment established by the apical endfeet is crucial for maintaining neuroepithelial (NE)/aRGs, proper timing of the detachment of the apical endfeet is critical for the quantitative control of neurogenesis in cerebral development. During delamination, the microtubule–actin–AJ (adherens junction) configuration at the apical endfeet shows dynamic changes, concurrent with the constriction of the AJ ring at the apical endfeet and downregulation of cadherin expression. This process is mediated by transcriptional suppression of AJ-related molecules and multiple cascades to regulate cell adhesion and cytoskeletal architecture in a posttranscriptional manner. Recent advances have added molecules to the latter category: the interphase centrosome protein AKNA affects microtubule dynamics to destabilize the microtubule–actin–AJ complex, and the microtubule-associated protein Lzts1 inhibits microtubule assembly and activates actomyosin systems at the apical endfeet of differentiating cells. Moreover, Lzts1 induces the oblique division of aRGs, and loss of Lzts1 reduces the generation of outer radial glia (oRGs, also called basal radial glia, bRGs), another type of neural progenitor cell in the subventricular zone. These findings suggest that neurogenic cell delamination, and in some cases oRG generation, could be caused by a spectrum of interlinked mechanisms.

## Introduction

**Figure 1:**
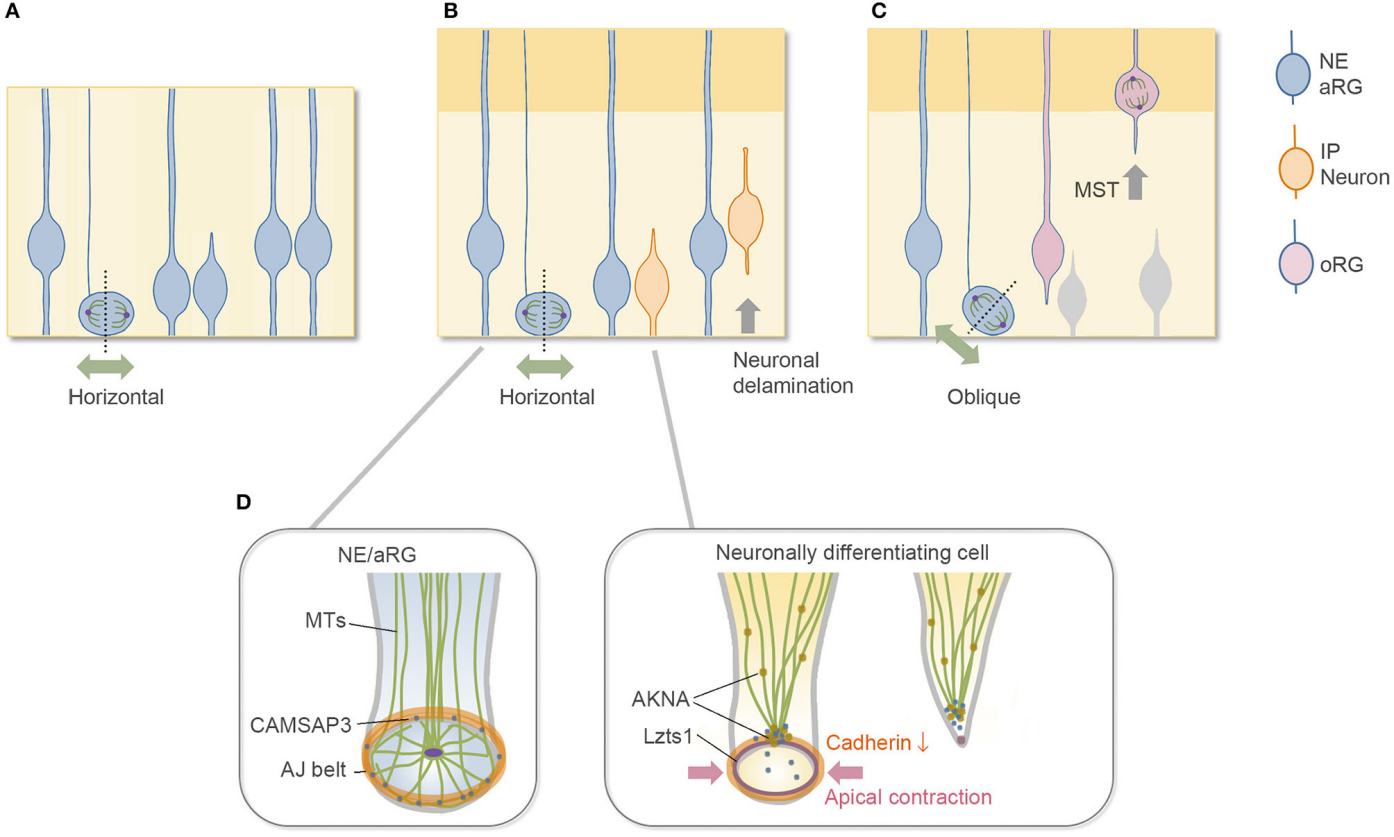
Division mode of aRGs and cytoskeletal architecture of apical endfoot. **(A)** Symmetric proliferative division of aRG at the early embryonic stage. **(B)** Neurogenic asymmetric division of aRG at the mid-embryonic, neurogenic stage. Most aRGs divide horizontally to allow both daughter cells to inherit the apical membrane. The neuronally differentiating daughter cell detaches its apical endfoot and starts to migrate basally (neurogenic cell delamination). **(C)** oRG-generating oblique division. The newly generated basal daughter cell does not inherit the apical junctional complex and migrates to the SVZ to become oRGs. **(D)** Cytoskeletal remodeling in the detachment of the apical endfoot during neurogenic cell delamination and its regulators. NE, neuroepithelial cell; aRG, apical radial glial cell (apical progenitor cell); IP, intermediate progenitor cell; oRG, outer radial glial cell (basal radial glial cell); MST, mitotic somal translocation; MTs, microtubules.

The vertebrate central nervous system originates from the neuroepithelium lining the embryonic neural tube. Neuroepithelial (NE) cells have polarized morphology along the radial axis, spanning the apical surface to the basal side at the basement membrane, and behave as neural progenitor cells. In the early period of mammalian cerebral wall development, neural progenitor cells (NE cells) undergo symmetric, proliferative division to expand the progenitor pool (Figure 1A). In the neurogenic period, the primary type of neural progenitor cell is called the apical radial glial cell, or aRG (also called apical progenitor cells, APs) (Miyata et al., 2001; Noctor et al., 2001; Uzquiano et al., 2018). Along with the progression of the cell cycle, aRGs undergo interkinetic nuclear migration (INM) in the ventricular zone (VZ) and divide at the apical surface (Figure 1B) to generate cells that differentiate to become an ordered series of neuron types. These differentiative aRG divisions are mostly asymmetric in terms of daughter cell fate; i.e., an aRG division generates one aRG and one neuronally differentiating cell, which are neurons for direct neurogenesis or intermediate progenitor cells (IPs) for indirect neurogenesis (Delaunay et al., 2017; Uzquiano et al., 2018). IPs have limited proliferative potential in rodent and typically undergo terminal mitosis to produce a pair of neurons in the subventricular zone (SVZ) (Haubensak et al., 2004; Miyata et al., 2004; Noctor et al., 2004). In mouse embryos, indirect neurogenesis substantially contributes to cortical expansion (Kowalczyk et al., 2009; Vasistha et al., 2015; Cárdenas et al., 2018). In both (direct and indirect) cases, these differentiative divisions typically occur horizontally along the apical surface with a cleavage along the apicobasal axis (Kosodo et al., 2004; Konno et al., 2008; Uzquiano et al., 2018), through which they inherit the apical membrane at birth (Shitamukai et al., 2011). Then, the newborn, neuronally differentiating daughter cells retract their apical processes to delaminate from the cadherin-based adherens junction (AJ) belt (Hatta and Takeichi, 1986) that packs the apical endfeet of VZ cells together (Figure 1B). When the daughter cell is a neuron, this delamination is the first step of neuronal migration, by which the daughter cells escape from the influence of extracellular cues at the apical side of the VZ.

This review article briefly describes the subcellular architecture of the apical endfeet, which provides an environment for proper neurogenesis from aRGs, and then summarizes our current knowledge on the molecular mechanisms underlying delamination. This review further discusses the common features of neurogenic cell delamination and outer radial glial cell (oRG) generation. oRGs, also called basal radial glial cells (bRGs), are another type of undifferentiated neural progenitor cell with long radial fibers extending to the basal side, and their cell body exists in the SVZ, where they divide multiple times (Fietz et al., 2010; Hansen et al., 2010; Wang et al., 2011; Pilz et al., 2013; Uzquiano et al., 2018). oRGs are first generated from aRGs, typically by oblique division at the apical surface (Shitamukai et al., 2011; LaMonica et al., 2013; Martínez-Martínez et al., 2016), and they migrate to the SVZ without inheriting the apical structure (Figure 1C). In this sense, oblique division is another step for daughter cells to disconnect and depart from the apical surface in addition to neurogenic cell delamination. Although the typical, major division patterns are summarized in Figures 1A–C, a relatively low proportion of neuronally differentiating cells may be generated by the oblique division in the rodent brain (Kosodo et al., 2004; Shitamukai et al., 2011), and it is unclear whether oRGs can be generated by the direct detachment of the apical processes. The relationship between the division angle of aRGs and their daughter cell fate is relatively complicated with differences at different developmental stages and in different species (Shitamukai and Matsuzaki, 2012; Gertz et al., 2014; Uzquiano et al., 2018).

Many studies have shown that the apicobasal (AB) polarity of aRGs is important for the maintenance of neural progenitor cells (or aRGs). Impaired AB polarity or apical protein complexes of aRGs induce cell cycle exit, precocious neuronal differentiation, and pathological delamination (Stocker and Chenn, 2009; Zhang et al., 2010; Hatakeyama et al., 2014; Camargo Ortega et al., 2019). This review does not discuss in detail AB polarity and its perturbations in neurodevelopmental disorders, as there are excellent reviews regarding these topics (Singh and Solecki, 2015; Arai and Taverna, 2017; Uzquiano et al., 2018; Hakanen et al., 2019).

## Apical Cytoskeletal Architecture Maintains Neural Progenitor Cells

The apical surface of the developing brain walls is formed by the apical endfeet of NE/aRG cells or VZ cells, which are tightly connected to each other by AJs with the cell adhesion molecule cadherin (Hatta and Takeichi, 1986; Nagasaka et al., 2016; Veeraval et al., 2020). The actin cytoskeleton is selectively concentrated and forms a dense and dynamic filament belt to support AJs of the apical endfeet (Lian and Sheen, 2015; Veeraval et al., 2020). The pharmacological inhibition of actomyosin at AJs reduces the concavity (Shinoda et al., 2018) and the stiffness (Nagasaka et al., 2016) of the apical surface, indicating that the actomyosin system contributes to these properties. Microtubule-based cellular organelles, such as centrosomes and primary cilia, are also positioned at the apical side of the NE/aRGs and are important for their morphology and cellular dynamics (Uzquiano et al., 2018; Park et al., 2019; Meka et al., 2020; Shao et al., 2020). Furthermore, the CAMSAP3 protein, which anchors non-centrosomal microtubules at the adhesion belt of cadherin-based AJs in epithelial cells (Meng et al., 2008), is also enriched at the AJs of the apical endfeet in the developing cortex (Camargo Ortega et al., 2019). These cytoskeletal architectures form a complex configuration at the apical endfeet (Figure 1D). In the NE cells of the chick spinal cord, a centrosome-nucleated wheel-like microtubule configuration aligns with the apical actin cable and AJs (Kasioulis et al., 2017), and a similar microtubule ring and intricate organization of the centrosome have been reported in the aRGs of the developing mammalian cortex (Shao et al., 2020).

These apical cytoskeletal architectures provide the environment for the proper proliferation and maintenance of NE/aRG cells. For example, from the apical surface, the cells receive signaling by soluble factors, such as epidermal growth factor (EGF), fibroblast growth factor (FGF), Neuregulin, and Shh, from cerebrospinal fluid (CSF) filling with the ventricle (Ferent et al., 2020). The direct physical contact of the apical endfeet provides the niche for activating Wnt–β-catenin signaling at the Cdh2 (N-cadherin) complex (Zhang et al., 2010) and Notch signaling (Hatakeyama et al., 2014). Additionally, Shao et al. showed that apical centrosome-organized microtubules maintain proper stiffness or tension of the apical membrane, which regulates aRG proliferation and neurogenesis through activation of YAP, a transcriptional coactivator in the HIPPO signaling pathway (Shao et al., 2020).

## Dynamic Cytoskeletal and AJ Remodeling in Cell Delamination

Neuronally differentiating cells generated by the horizontal division of aRGs inherit the apical membrane at birth, and then, they detach their apical endfeet from the cadherin-based AJ belt. Upon this delamination, the microtubule–actin–AJ cytoskeletal architecture at the apical endfeet shows dynamic changes (Das and Storey, 2014; Kasioulis et al., 2017; Camargo Ortega et al., 2019), concurrent with the constriction of the AJ ring at the apical endfeet and downregulation of cadherin expression at the AJs (Figure 1D). The constriction of the apical AJ ring primarily occurs by activation of the actomyosin system. In the chick spinal cord, this apical constriction allows the delaminating neurons to leave behind their apical tip with the primary cilia (“apical abscission”). Then, the primary cilia are rapidly reassembled in the differentiating neurons during the apical process retraction. These cilium dynamics may switch the Shh signaling pathway from canonical to noncanonical (Das and Storey, 2014; Kasioulis et al., 2017; Toro-Tapia and Das, 2020). In mice, the apical plasma membrane protrusions of the NE cells and Prominin-1 (CD133)-enriched extracellular membrane particles in the ventricular fluid were observed (Dubreuil et al., 2007; Corbeil et al., 2010), providing the possibility that the apical abscission-like phenomenon might also occur in the developing cerebrum. Unlike in the chick neural tube, however, the apical abscission that leaves behind the primary cilia (Das and Storey, 2014) has not been reported yet in the developing mouse brain; instead, the basolateral cilia are formed by nascent differentiating cells before delamination (Wilsch-Bräuninger et al., 2012; Tozer and Morin, 2014). Such basolateral cilium possibly reduces the exposure to luminal mitogen such as Shh (Arai and Taverna, 2017), but the experimental loss of primary cilia after around embryonic day (E) 11 in mice does not alter cortical neurogenesis (Shao et al., 2020). Overall, these results suggest the evolutionarily or regionally different cilium dynamics and functions in the delamination and early differentiation steps.

## Apical Detachment and Neurogenesis

Since the environment established by the subcellular architecture at the apical endfeet is crucial for maintaining the NE/aRGs as described above, the experimentally induced detachment of the apical processes of the cells sometimes promotes the differentiation cascade in the rodent brain (Arimura et al., 2020). For example, if Cdh2 expression is experimentally eliminated *in vivo*, abnormal rapid delamination and differentiation of aRGs are observed (Zhang et al., 2010; Hatakeyama and Shimamura, 2019). Furthermore, as nascent differentiating cells express Dll1, a ligand of Notch signaling, at their apical endfeet, their detachment itself changes the microenvironment around the cells during delamination (Kawaguchi et al., 2008; Hatakeyama et al., 2014). If the apical endfeet retention period before delamination is experimentally lengthened, neuronal production from aRGs is decreased during a certain period (Hatakeyama and Shimamura, 2019). These observations suggest that proper detachment timing of the apical endfeet is critical for the quantitative control of neurogenesis in cerebral development.

In physiological scenarios, however, the inheritance of the apical epithelial structure or detachment of apical endfeet themselves seems not to determine the daughter cell's identity (neuronally differentiating or undifferentiating) in neocortical development. For example, at the early developmental stage, during which NE cells undergo symmetric proliferative division, both daughter cells retain the apical endfeet (Figure 1A), and if one cell becomes detached from the apical surface during division, it regenerates the apical endfeet (Fujita et al., 2020). This phenomenon contributes to the robust epithelial structure at the early stage but is not observed in daughter cells during the neurogenic stages. Another example is the oRG generation, in which the daughter cells to become oRGs are detached from the apical surface but still undifferentiated. In addition to the basal processes, the cell intrinsic and extrinsic cues contribute to the maintenance and proliferation of the oRGs in a species-different manner (Tsunekawa et al., 2012; Uzquiano et al., 2018; Penisson et al., 2019; Kalebic and Huttner, 2020).

## Molecules Linking Commitment and Delamination

Cell delamination is the dynamic event with cytoskeletal remodeling of the apical microtubule–actin–AJ configuration (Kasioulis et al., 2017). This step is mediated by transcriptional suppression of AJ-related molecules and multiple cascades to regulate cell adhesion and cytoskeletal architecture in a post-transcriptional manner (Camargo Ortega et al., 2019; Kawaue et al., 2019; Arimura et al., 2020). Moreover, knockdown of cell-surface molecule TAG-1 results in the retraction of the basal processes of progenitors, which induces overcrowding of the subapical region to evoke cell departures with retraction of the apical processes. This observation suggests passive forces from neighboring crowding cells also regulate the departure of cells (Okamoto et al., 2013). These redundant regulatory mechanisms of delamination will contribute to robust brain histogenesis.

Recent advances have added to the molecules that link neuronal commitment and delamination as below.

### Transcription Factors

Since fate decisions of daughter cells likely occur prior to or during cell division of aRGs (Uzquiano et al., 2018), neuronal commitment is thought to proceed before detachment of the apical endfeet in one of the daughter cells in the case of neurogenic asymmetric division (Figure 1B): thus, proneural gene(s) expression is a candidate for the switch that starts the delamination cascades. The proneural genes Neurogenin 2 (Neurog2) and Ascl1 activate the Rho GTPases Rnd2 and Rnd3, respectively, to reorganize the actin cytoskeleton by inhibiting Rho activity in migrating neurons (Ge et al., 2006; Heng et al., 2008; Pacary et al., 2011); therefore, these proneural genes might also be implicated in delamination by modulating the cytoskeleton.

Neurog2 and several transcription factors downstream of Neurog2 are reported to be involved in delamination through transcriptional suppression of cadherins and AJ-related molecules (Pacary et al., 2012; Itoh et al., 2013b; Singh and Solecki, 2015). The overexpression of Neurog2 represses Cdh1 (E-cadherin) transcription in cultured cortical neural progenitor cells (Itoh et al., 2013a). In the spinal cord, Foxp2 and Foxp4, known as transcriptional repressors, promote neuronal delamination through direct transcriptional suppression of Cdh2, and Foxp4-mutant and Foxp-misexpression studies suggest similar functions of these molecules in delamination in the developing cortex (Rousso et al., 2012). Tbr2 (Eomes) promotes the detachment of cells from the apical surface and their differentiation (Sessa et al., 2008). Tavano et al. showed that another transcription factor, insulinoma-associated 1 (Insm1), is upregulated by Neurog2 in neuronal commitment and promotes delamination by repressing the AJ belt-specific protein Plekha7 (Farkas et al., 2008; Tavano et al., 2018; Kalebic and Huttner, 2020). The epithelial–mesenchymal transition (EMT)-related transcription factors Scratch1 and Scratch2, members of the Snail superfamily, are also expressed upon neuronal fate commitment by upregulation of proneural genes such as Neurog2 and induce delamination by transcriptional repression of the adhesion molecule Cdh1 (Itoh et al., 2013a).

### Slit-Robo Signal

In the developing cerebral cortex, the absence of Robo receptors (Robo1/2 mutant) decreases Hes1 messenger RNA (mRNA) levels and produces an excess of IPs (Borrell et al., 2012; Cárdenas et al., 2018). Interestingly, a large proportion of Robo1/2 mutant Ips fail to retract their apical processes from the apical surface. This mutant phenotype is accompanied by enhanced thickness of the apical band in Cdh2 and ß-Catenin immunoreactivity (Borrell et al., 2012). Thus, Robo signaling inhibits cadherin-based adhesions at apical processes, similar to retinal ganglion cells (Wong et al., 2012), whereas its molecular link to the cytoskeletal architecture of apical Ajs is still unknown.

### AKNA

Recently, Camargo Ortega et al. reported that the centrosome protein AKNA is localized at the interphase centrosome of neuronally differentiating cells and SVZ progenitors in the developing cerebrum at the neurogenic stage (Camargo Ortega et al., 2019). The authors further demonstrated that AKNA overexpression induced rapid delamination, and conversely, AKNA loss-of-function impairs delamination, indicating that AKNA plays a crucial role in delamination. The delamination processes are primarily mediated by AKNA's effect on microtubule dynamics that destabilize apical microtubule–actin–AJ complexes, which promote constriction of the apical endfeet (Camargo Ortega et al., 2019).

In TGFb1-treated murine mammary gland epithelial (NMuMG) cells during EMT, AKNA recruits the microtubule minus-end binding protein CAMSAP3 (Tanaka et al., 2012) from junctional microtubules to the centrosome (Camargo Ortega et al., 2019), suggesting that this molecular mechanism underlying EMT (Pongrakhananon et al., 2018) also regulates delamination in neocortical development (Figure 1D). Moreover, a transcription factor SOX4, which regulates EMT of NMuMG cells (Tiwari et al., 2013), upregulates *Akna* mRNA in NMuMG cells in EMT and neural stem cell line N2A cells (Camargo Ortega et al., 2019), and SOX4 overexpression generates SVZ progenitors in the developing brain (Chen et al., 2015). These observations further support that AKNA regulates neurogenic cell delamination through EMT-like molecular mechanisms.

### Lzts1

Our research group has recently found that leucine zipper putative tumor suppressor 1 (Lzts1) (also known as FEZ1 and PSD-Zip70) (Konno et al., 2002) acts as a master modulator of neurogenic cell delamination (Kawaue et al., 2019). Lzts1 is reported as a microtubule-associated protein that inhibits microtubule polymerization (Ishii et al., 2001) and is implicated in several human cancers (Vecchione et al., 2007). Notably, Lzts1 expression is upregulated by Neurog1/2 and closely localizes at the AJ belts of the apical processes of differentiating newborn cells (Kawaguchi et al., 2008; Kawaue et al., 2019). Overexpression of Lzts1 induces apical contraction with a decrease in the expression of Cdh2 at AJs, which results in detachment of the apical processes. In contrast, loss of Lzts1 impairs the differentiating cells from departing the apical surface. Thus, local Lzts1 expression at endfeet AJs has a unique function that positively controls neurogenic cell delamination in the developing cortex.

Lzts1-induced apical contraction is mediated by activation of the actomyosin system (Kawaue et al., 2019), whereas apical contraction by the activation of myosin II does not solely reduce cadherin expression and is not sufficient to induce detachment (Das and Storey, 2014). Therefore, the function of Lzts1 in delamination is likely caused by the coordinated cytoskeletal rearrangement of the microtubule–actin–AJ complex at the apical endfeet mediated by both inhibiting microtubule polymerization and activating actomyosin systems (Kawaue et al., 2019) (Figure 1D).

### DSCAM

In the mouse dorsal midbrain, down syndrome cell adhesion molecule (DSCAM) has been shown to control neuronal delamination. DSCAM starts to be expressed in differentiated neurons only before migration and locally suppresses the RapGEF2–Rap1–Cdh2 cascade at their apical endfeet to delaminate (Arimura et al., 2020).

## Common Mechanisms in Neurogenic Cell Delamination and oRG Generation

**Figure 2:**
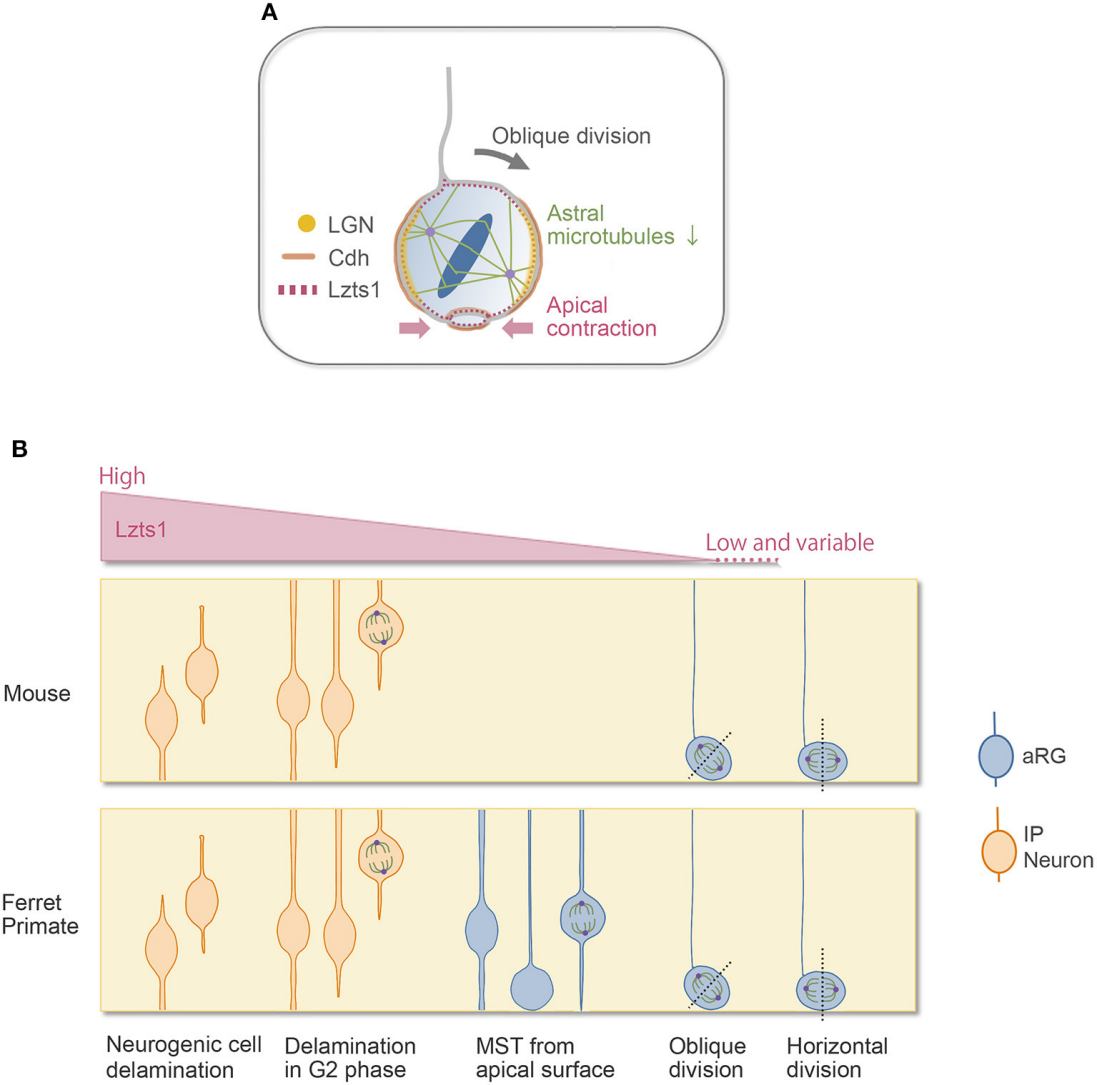
Lzts1 controls both neuronal and progenitor cell delamination. **(A)** Weak Lzts1 expression induces oRG-generating oblique division by inhibiting centrosome anchoring to the lateral side in mitosis (model). The apical contraction induced by Lzts1 may also contribute to oblique division. Lzts1 induces MST of basal daughter cells by activating the actomyosin system. **(B)** Lzts1 controls both neurogenic cell delamination and oRG generation as a master modulator of the cytoskeleton. In the developing cerebrum, aRGs/IPs show various behaviors to generate their daughter cells. In addition to the typical detachment of the differentiating daughter cells (Figure 1B), some IP cells shed their apical processes during G2 and then show MST and divide in the SVZ. There is also a rare pattern in human and ferret oRG generation, where MST occurs from the apical surface (Gertz et al., 2014). Experimental Lzts1 expression levels correlate with these diverse cellular behaviors. *In vivo*, Lzts1 is expressed at high levels in neuronally differentiating cells, including nascent neurons and IPs, whereas in the aRG, Lzts1 exhibits variable and weak expression.

oRGs can be produced by the oblique (or perpendicular) cell divisions of aRGs (LaMonica et al., 2013; Gertz et al., 2014; Martínez-Martínez et al., 2016). With the oblique division, the newly generated basal daughter cells do not inherit the apical junctional complex and can migrate to the SVZ to become oRGs (or oRG-like cells) (Figures 1C, 2A). Even though they lack apical anchoring, these basal daughter cells still have proliferative potential, and their basal processes are considered a key morphological feature underlying this capacity (Tsunekawa et al., 2012; Uzquiano et al., 2018; Kalebic and Huttner, 2020). Many genes and extracellular factors contribute to the amplification of oRGs in the SVZ, and some oRG-specific genes that are present only in humans or primates, such as ARHGAP11B, are thought to explain the evolutional expansion of the neocortex (Florio et al., 2015; Penisson et al., 2019).

The molecular mechanisms regulating oRG generation at the apical surface have been partially uncovered. In the VZ during the restricted period for massive oRG generation, *Cdh1* mRNA is expressed at a significantly lower level than that during the other periods. Reduced Cdh1 function increases oRG generation by both weakening cell adhesion and promoting oblique division in the ferret brain (Martínez-Martínez et al., 2016). Furthermore, in the epithelial cells, the cell division orientation is shown to be coupled to cell–cell adhesion by the LGN–Cdh1 complex (Gloerich et al., 2017). These evidences suggest that AJ-related molecules are involved in the regulation of spindle orientation in oRG generation.

RNA-seq analysis suggested that neuronally differentiating cells and some oRGs might share common molecular features (Johnson et al., 2015), and forced Neurog2 expression in the ferret brain induced the generation of oRG-like cells *in vivo* (Johnson et al., 2015). These observations raise an intriguing possibility that proneural genes or delamination cascades may underlie the generation of a subset of oRGs.

In line with this, we found that Lzts1, a key molecule of neurogenic cell delamination, also induces oRG generation by the oblique division of aRGs (Kawaue et al., 2019). Single-cell analysis (Okamoto et al., 2016) shows that in the E14 mouse VZ, when oRG-like cells are generated from aRGs, some aRGs weakly express *Lzts1* mRNA. Weakly forced-expressed Lztz1 localizes to the cell cortex of aRGs in mitosis and induces oblique division. Conversely, loss of Lzts1 decreases the oblique division frequency in mice and reduces oRG generation in mice and ferrets. Currently, the precise molecular mechanisms underlying Lzts1-mediated oblique division are unclear. Live imaging of the Lzts1-expressed aRG suggests that Lzts1 inhibits the anchoring of centrosomes to the subapical (basolateral) portion of the process during M phase (Kawaue et al., 2019) (Figure 2B). On the other hand, the basolateral localization of LGN, which binds Numa to orient the mitotic spindle by anchoring spindle astral microtubules (Konno et al., 2008), is maintained in the Lzts1-induced obliquely dividing aRGs, suggesting that the localized LGN–Cdh complex might be relatively maintained. Since Lzts1 has inhibitory effect on the microtubule assembly (Ishii et al., 2001), low-level Lzts1 in mitotic aRGs may perturb the formation of astral microtubules and inhibit the astral microtubule–LGN–AJ interaction, which may induce oblique division (Kawaue et al., 2019) (Figure 2A). Consistently, Btg2::GFP+ neuronal progenitors, which should express *Lzts1* mRNA (Kawaguchi et al., 2008; Schenk et al., 2009), show more variable spindle orientation with relatively small astral microtubules than those of proliferating progenitors (Mora-Bermúdez et al., 2014). Moreover, the function of Lzts1 on apical contraction may also be involved in inducing oblique division (Kawaue et al., 2019) (Figure 2A). The latter mechanism might link the apical process retraction with the spindle orientation change in some experimental conditions manipulating a certain number of genes (Lancaster and Knoblich, 2012; Mora-Bermúdez and Huttner, 2015).

Overall, these observations suggest that, in the case of Lzts1, the oblique division that generates oRGs is controlled by a molecular mechanism similar to that of delamination in the context of the microtubule–AJ complex. Therefore, the junctional proteins would play critical roles both in maintaining epithelial structure at the apical endfeet (Zhang et al., 2010; Veeraval et al., 2020) and, as in the case of the epithelial cells (Gloerich et al., 2017), in controlling the spindle orientation in aRGs. It is an open question whether the adhesion molecules, Cdh1 and Cdh2, differently play these two roles in aRGs.

Lzts1 function in oblique division may explain some of the diverse, contradictory conclusions of previous studies on spindle orientation and fate determinant (Lancaster and Knoblich, 2012; Mora-Bermúdez and Huttner, 2015): if the experimentally manipulated molecules have some functions in the maintenance of aRGs, their depletion increases the expression levels of neuronal molecules (molecules upregulated under proneural transcription factors) including Lzts1 in the dividing aRGs, which will increase the frequency of oblique division. This interpretation may explain why the oblique or perpendicular divisions of aRGs are correlated with the progenies' neuronal fate under some experimental conditions, which is distinct from the physiological situation in which most differentiative divisions occur horizontally (Shitamukai and Matsuzaki, 2012; Uzquiano et al., 2018). If the experimental conditions have no or weak effect on the maintenance of aRGs but strongly impair the apical AJ complex, aRGs would detach or delaminate without neuronal differentiation.

## A Continuous Spectrum of Mechanisms Controlling Delamination

Unlike AKNA, which primarily affects microtubule dynamics (Camargo Ortega et al., 2019), Lzts1 activates actomyosin systems in addition to its inhibitory effect on microtubules. The activating effect of Lzts1 on the actomyosin system does not seem to require its inhibitory effect on microtubule assembly because cellular stiffness measurement by atomic force microscopy (AFM) reveals that in Lzts1-overexpressing NIH3T3 cells, even under Taxol (microtubule stabilizer) treatment, Lzts1 increases cellular stiffness by activating myosin II (Kawaue et al., 2019). Furthermore, live imaging of the Lzts1-expressing cerebral walls shows that Lzts1 strongly induces mitotic somal translocation (MST), in which the soma rapidly translocates basally before cytokinesis (Kawaue et al., 2019). MST is the characteristic behavior observed in oRG or IP migration (Hansen et al., 2010; Gertz et al., 2014; Ostrem et al., 2014) (Figure 1C). MST requires the activation of the Rho–ROCK–myosin II pathway but not microtubule motors or centrosomal guidance (Ostrem et al., 2014, 2017).

In normal neocortical development, there are various cell departure patterns from the apical surface in the developing cerebral wall, as shown in Figure 2B. Interestingly, these diverse cellular behaviors appeared in response to the level of overexpressed Lzts1, suggesting that the various cellular departure events might be understood as a continuous phenomenon linked to common molecular mechanisms, likely as a spectrum (Kawaue et al., 2019) (Figure 2B). Further research is needed to elucidate the precise molecular mechanisms by which Lzts1 orchestrates cytoskeletal dynamics to induce neuronal differentiation, MST, and oRG generation in neocortical development.

In mice with lissencephalic brains, the number of oRGs is small, and their self-renewal potential in the SVZ is relatively limited (Wang et al., 2011) (thus, sometimes they are interpreted as “oRG-like” cells). In contrast, in species with gyrencephalic brains, such as ferrets and primates, oRGs are more abundant and self-renew, producing many IPs and neurons (Hansen et al., 2010; García-Moreno et al., 2012; Reillo and Borrell, 2012; Betizeau et al., 2013; Gertz et al., 2014). The unique cellular behaviors related to oRG generation, i.e., oblique division, and MST show evolutionary changes in their frequency and distance (LaMonica et al., 2013; Ostrem et al., 2014, 2017). Lzts1 expression is weak and variable in the aRG population in mice, and its expression levels are likely regulated by the oscillatory/variable expression of Hes1 and proneural genes (Shimojo et al., 2008; Kawaue et al., 2019; Kageyama et al., 2020). Since it is still unknown whether the differential expression of Lztz1 in neural progenitor cells might be involved in the differential cell behaviors between species, it would be interesting to address this question in the future.

## Author Contributions

AK wrote and edited the manuscript.

## Conflict of Interest

The author declares that the research was conducted in the absence of any commercial or financial relationships that could be construed as a potential conflict of interest.
